# Impact of a patient-flow physician coordinator on waiting times and length of stay in an emergency department: A before-after cohort study

**DOI:** 10.1371/journal.pone.0209035

**Published:** 2018-12-14

**Authors:** Marta Morais Oliveira, Christophe Marti, Majd Ramlawi, François P. Sarasin, Olivier Grosgurin, Pierre-Alexandre Poletti, Frédéric Rouyer, Olivier T. Rutschmann

**Affiliations:** 1 Geneva School of Medicine, Geneva, Switzerland; 2 Division General Internal Medicine, Department of Internal Medicine, Rehabilitation and Geriatrics, Geneva University Hospitals and School of Medicine, Geneva, Switzerland; 3 Division of Emergency Medicine, Department of Community, Primary Care and Emergency Medicine, Geneva University Hospitals and School of Medicine, Geneva, Switzerland; University of Malta Faculty of Health Sciences, MALTA

## Abstract

**Objective:**

Overcrowding is common in most emergency departments (ED). Despite the use of validated triage systems, some patients are at risk of delayed medical evaluation. The objective of this study was to assess the impact of a patient-flow physician coordinator (PFPC) on the proportion of patients offered medical evaluation within time limits imposed by the Swiss Emergency Triage Scale (SETS) and on patient flow within the emergency department of a teaching urban hospital.

**Methods:**

In this before-after retrospective cohort study, we compared the proportions of patients who received their first medical contact within SETS-imposed time limits, mean waiting times before first medical consultation, mean length of stay, and number of patients who left without being seen by a physician, between two periods before and after introducing a PFPC. The PFPC was a senior physician charged with quickly assessing in the waiting area patients who could not immediately be seen and managing patient flow within the department.

**Results:**

Before introducing the PFPC position, 33,605 patients were admitted, versus 36,288 after. Introducing a PFPC enabled the department to increase the proportion of patients seen within the SETS-imposed time limits from 60.1% to 69.0% (p <0.0001). Waiting times until first medical consultation were reduced on average by 27.7 minutes (95% confidence interval [95% CI]: 25.9–29.5, p < .0001). No significant differences were observed as to length of stay or number of patients who left without being seen between the two study periods.

**Conclusions:**

Introducing a physician dedicated to managing patient flow enabled waiting times until first medical consultation to be reduced, yet had no significant benefit for patient flow within the ED, nor did it reduce the number of patients who left without being seen.

## Introduction

Overcrowding is common in most emergency departments (EDs), due to imbalance between patient input, ED throughput and patient output [1]. This may negatively impact patient & staff satisfaction and quality of care [2].

Different triage systems have been implemented in order to quickly identify high-risk patients upon arrival and to put the right patient, in the right place in the optimal timeframe. In our ED, all patients are triaged and categorized into four risk levels using the Swiss Emergency Triage Scale (SETS) [3–5]. In adherence to the SETS, the first medical assessment should be performed immediately for Level 1 emergencies, within 20 minutes for Level 2, and within 120 minutes for Level 3. Despite significant efforts to improve patient flow, in our ED more than 40% of patients classed as Level 2 or 3 have to wait longer than 20 and 120 minutes respectively.

Different studies have shown that a rapid medical evaluation at triage was able to reduce waiting times before medical assessment, length of stays in the ED, and the number of patients who leave before consultation [6–10]. This is why the role of a patient-flow physician coordinator (PFPC) was created in our ED.

The study primary objective was to evaluate the impact of a PFPC on the proportion of patients having medical assessment within SETS-imposed time limits. Its secondary objectives were to evaluate PFPC's impact on waiting times until medical assessment, length of stay in the ED, and on the number of patients leaving without being seen.

## Methods

### Study design

This was a retrospective before-after cohort study, with data on patient flows before PFPC introduction compared to those after PFPC introduction.

### Study setting

The ED of Geneva University Hospitals, primary and tertiary teaching urban hospital, admits 64,000 patients every year. After triage, patients are evaluated in the Emergency room for the most acute cases, in the Urgent care sector for the less severe cases and in the Psychiatric emergency sector for patients with psychiatric emergencies.

### Study population

Inclusion criteria concerned all adult patients (≥16 years old) admitted to Geneva University Hospitals’ ED between April 1, 2014, and March 31, 2016, triaged to the Emergency room or to its waiting room. Exclusion criteria concerned patients admitted to the ED and triaged to other emergency sectors (urgent care or psychiatric care).

### Patient-flow physician coordinator

The PFPC was an experienced senior emergency physician, who was present in the triage and waiting area from 7:30 AM to 10:30 PM from Monday to Friday, responsible for (1) conducting a quick medical evaluation of patients who could not immediately be admitted to the emergency room and were first admitted to its waiting room, (2) prioritizing or redirecting waiting patients, (3) prescribing laboratory tests or X-rays, and (4) managing patient flow within the emergency room.

### Data collection

All data were extracted from electronic medical and administrative records. In order to fulfill the primary objective, the proportions of patients admitted within SETS-fixed time limits were compared between two study periods (2014–2015 *vs*. 2015–2016) for Level 1–3 SETS-classed patients. As no minimum evaluation time limit is defined by the SETS for Level 4 cases, these were not included in the analyses. To fulfill the secondary objectives, the following data were collected and compared between the two study periods: waiting times until first medical evaluation, length of stay in the Emergency room, and number of patients who left before receiving medical assessment.

As the PFPC was only present from 7:30 AM to 10:30 PM from Monday to Friday, secondary analyses were also planned for both the primary and secondary objectives in order to limit analyses to the patients admitted during the times the PFPC was present in the ED.

### Statistics

Data for the periods before and after introducing the PFPC were compared using Student’s t-tests for continuous variables and Chi-squared tests for categorical variables, using IBM SPSS Statistics software for Windows, Version 22.0, Armonk, NY.

### Ethics approval and trial registration

The study protocol was approved by our local ethics committee (*Commission Centrale d’Ethique et de la Recherche du Canton de Genève*). The ethics committee waived the investigators from informed consent due to the retrospective nature of the study and the use of fully anonymised data. Trial registration: ClinicalTrials.gov identifier: NCT02980159

## Results

### Study population

From April 1, 2014, to March 31, 2015, or the period before introducing the PFPC, a total of 33,605 patients were admitted to the Emergency room and its waiting room, compared to 36,288 in the period of April 1, 2015, to March 31, 2016, or after introducing the PFPC, *i*.*e*., an 8% between-group increase in the number of patients admitted. No significant differences were observed between these populations (Table 1).

**Table 1:** Characteristics of patients admitted before and after introducing a patient-flow physician coordinator.

	Before PFPC (n = 33, 605)	After PFPC (n = 36,288)
Age, mean (+/- SD)	56.7 (22.2)	56.7 (22.3)
Male gender, n (%)	17,845 (53.1%)1	19,040 (52.5%)
SETS emergency level,n (%)		
• 1	4.437 (13.2%)	4,838 (13.3%)
• 2	14,770 (44%)	16,042 (44.2%)
• 3	13,881 (41.3%)	14,803 (40.8%)
• 4	517 (1.5%)	605 (1.7%)

PFPC, patient-flow physician coordinator; SD, standard deviation; SETS, Swiss Emergency Triage Scale.

### Primary objective

Introducing a PFPC resulted in a significant increase in the proportion of patients having their first medical evaluation within the SETS-fixed time limits, from 60.1% to 69.0% for all emergency levels, or a relative increase of 14.8% (Table 2). The presence of the PFPC resulted in a relative increase of 10.7% in the number of Level 2 patients receiving a first medical evaluation within 20 minutes of arrival, and a 25.6% increase in the number of Level 3 patients receiving a first medical evaluation within 120 minutes (Table 2).

**Table 2:** Number and proportions of patients evaluated within SETS time limits before and after introducing the patient-flow physician coordinator.

	All Patients			Patients admitted during the times the PFPC was present*		
	Before PFPC	After PFPC	p	Before PFPC	After PFPC	p
SETS emergency level						
SETS, all levels, n (%)	19.888 (60.1)	24.638 (69)	<0.0001	9.947 (58.1)	12.692 (68.3)	<0.0001
SETS, Level 1, n (%)	3.927 (88.5)	4330 (89.5)	0.13	2.239 (87.4)	2428 (88.7)	0.15
SETS, Level 2, n (%)	7.899 (53.5)	9.500 (59.2)	<0.0001	4265 (52.6)	5.166 (58.5)	<0.0001
SETS, Level 3, n (%)	8.062 (58.1)	10.808 (73)	<0.0001	3.443 (53.4)	5.098 (72.6)	<0.0001

SETS, Swiss Emergency Triage Scale; PFPC, patient-flow physician coordinator.

*PFPC presences = 7:30 AM– 10:30 PM Monday to Friday.

By limiting the analyses to the periods of presence of the PFPC, the proportion of patients evaluated within the time limits increased from 58.1% to 68.3%. During these periods, we observed a relative increase of 11.2% and 36.0% for Level 2 and 3 cases, respectively (Table 2). During the hours when the PFPC was absent (nights and weekends), there was still a statistically-significant increase in the number of patients triaged as Level 2 and 3 who had a first medical evaluation within the SETS-fixed time limits. For Level 2 cases, the proportion of patients seen within the time limits increased from 54.6% before the PFPC was introduced to 60.1% after (p <0.0001). For Level 3 cases, this proportion increased from 62.1% to 73.7% (p <0.0001).

### Secondary objectives

After the introduction of a PFPC, a significant reduction in waiting times until first medical contact was observed, with overall decreases from a mean of 86.7 minutes to 59.0 minutes for all risk levels, *i*.*e*., a 27.7-minute decrease on average (95% confidence interval [95% CI]: 25.9–29.5). This decrease was 13.9 minutes (95% CI: 12.0–15.8) and 50.5 minutes (95% CI: 47.1–53.9) for Level 2 and 3 emergencies, respectively (Table 3).

**Table 3:** Waiting times until first medical contact, before and after introducing patient-flow physician coordinator.

Time until first medical contact	Before PFPC	After PFPC	p-value
All SETS levels			
• Mean in minutes (+/- SD)	86.7 (134.4)	59.0 (102.9)	<0.0001
• Median in minutes (IQR)	24 (0–120)	14 (0–74)	
SETS level 1			
• Mean in minutes (+/-SD)	6.1 (25.6)	5.6 (31.0)	0.42
• Median in minutes (IQR)	0 (0–0)	0 (0–0)	
SETS level 2			
• Mean in minutes (+/-SD)	55.3 (92.2)	41.4 (74.3)	<0.0001
• Median in minutes (IQR)	15 (0–74)	10 (0–52)	
SETS level 3			
• Mean in minutes (+/-SD)	144.2 (164.2)	93.7 (127.8)	<0.0001
• Median in minutes (IQR)	87 (14–223)	44 (6–131)	

SETS, Swiss Emergency Triage Scale; PFPC, patient-flow physician coordinator; SD, standard deviation; IQR,interquartile range.

Between the two study periods, no significant changes in length of stay in the Emergency room were observed. Similarly, the number of patients who left without being seen remained stable (Table 4).

**Table 4:** Secondary objectives: Length of stay in the emergency room and proportion of patients who left without being seen, before and after introducing a patient-flow physician coordinator.

	Before PFPC	After PFPC	p
Length of stay in the Emergency Room (hours)			
Mean (+/-SD)	5.41 (3.23)	5.39 (3.37)	0.68
Median (IQR)	4.87 (3.35–6.77)	4.82 (3.37–6.70)	0.34
Patients who left without being seen, n (%)	2.184 (6.5)	2.330 (6.4)	0.67

SETS, Swiss Emergency Triage Scale; PFPC, patient-flow physician coordinator; SD, standard deviation; IQR, interquartile range.

## Discussion

Despite the 8% between-group increase in the number of patients admitted, the presence of a PFPC resulted in a 14.8% increase in the proportion of patients who had their first medical evaluation within the time limits imposed by the SETS. Although the PFPC had no impact on Level 1 emergencies, it resulted in mean reductions of 14 minutes in waiting times before medical evaluation for Level 2 emergencies, and of 50 minutes for Level 3 patients.

Different models aiming at reducing waiting times prior to the first medical evaluation have been evaluated. The majority of these studies have assessed the impact of a physician working directly with triage nurses. This assessment, combined with triage, enables waiting times until first medical consultation to be significantly reduced, as well as decreasing the number of waiting patients, which is its inherent purpose [8, 11–16]. In our model, similar to that described by other teams [9, 11, 17], the PFPC physician was not charged with triaging all the patients, but rather focused on patients in the waiting area who could not immediately be admitted to the Emergency room.

In contrast to previous studies [15], the effect on waiting times until medical evaluation remained significant even when the PFPC was absent, suggesting this position had a more systemic impact within our department. This could be due to a collective awareness as to the relevance of providing medical assessment more rapidly, even in the absence of the PFPC. Alternatively, the consistent positive effect observed on patient flow could result from the beneficial impact of when the PFPC was present in freeing up the waiting area, thus still enabling faster management and admission of arriving patients, even after his departure. Of note, no other reorganization or change in our processes were implemented during these time periods.

Despite reducing waiting times until first medical assessment, the PFPC had no effect on the ED’s length of stay. The impact of early medical assessment on length of stay in ED varies from study to study. Some studies have reported a positive impact on length of stay, suggesting that early assessment could help physicians anticipate certain tests, thus reducing time spent in the ED [7, 9, 11, 12, 16]. On the other hand, other studies have reported no significant impact on hospital lengths of stay, particularly for more severe cases, such as those included in our study [15, 17, 18]. Finally, one recent study reported that triage physicians are at risk of prescribing more radiological examinations, thereby causing a negative impact on patient flow and length of stays [13]. This risk of over-prescribing radiological examinations was also observed when a physician assessed patients in the waiting room [19].

The absence of any effect on ED’s length of stay in our population could be due to the fact that the PFPC’s role was mainly focused on the patients in the waiting area of the Emergency room. Patient flow within an ED is subject to different factors, such as waiting times for additional examinations or specialists’ consultations, as well as hospital bed availability. Given that the PFPC’s role did not focus on these elements, it is hardly surprising that lengths of stay in our Emergency room did not differ between the two study periods.

Finally, several studies have demonstrated that early medical assessment is crucial in reducing the risk of patients leaving without receiving medical evaluation [7, 9, 11, 12, 16, 17, 20]. In our study, the proportion of patients who left without being seen remained stable between the two periods. The retrospective design of our study did not allow us to identify the factors associated with this risk.

Our study has limitations. First, it was a nonrandomized retrospective single-center study comparing two study periods. This design underlines how difficult it is to perform randomized studies in this specific research domain, and thereby limits generalization. For these reasons, only a few randomized studies have tested the impact of a triage physician [9, 12, 15]. In our institution, the introduction of a PFPC was mandated by the direction of the hospital, precluding us from performing a randomized study. Moreover, the introduction of a PFPC may have a systemic impact which may continue in the non-intervention period in an interrupted time series design. Before-after studies are prone to potential confounding, and the observed reduction in time to first medical assessment might be explained by other factors than the intervention itself. However, no other organizational change than PFPC introduction occurred in our department between the two study periods. All consecutive patients over two consecutive years were included in order to reduce potential confounding. Age, gender and levels of emergency of included patients were similar over the two study periods but residual confounding cannot be excluded. Secondly, different triage systems and medical support models have been tested. The results observed in our SETS-based system, with one physician intervening immediately after triage, can thus not be directly applied to other contexts. Furthermore, the impact of our intervention on patient quality of care is difficult to evaluate. Quality of care cannot, in fact, be reduced to simply improving waiting times prior to medical evaluation. In order to explore this more precisely, other indicators of quality should have been investigated. Fourthly, the costs of our intervention were not evaluated. Partovi *et al*. reported costs of implementing this coordinating role exceeding a million dollars per year [20]. The cost-benefit ratio of such an intervention has never been assessed, and these excessive costs have led some EDs to stop the triage physician activity [18]. In our department, the role of the PFPC was performed by senior doctors with no additional resources. This could potentially run the risk of reducing the capacity of these physicians in supervising junior doctors within the ED, as well as the teaching they provide to them. It could thus negatively impact the overall quality of patient care, as well as training of future emergency physicians. Fifth, the satisfaction of patients and collaborators was not assessed in our study. Nevertheless, most physicians expressed informally that this role was somewhat laborious, while the nursing staff generally viewed it as useful and reassuring. These statements should be evaluated by a standardized questionnaire. Finally, the use of resources and tests prescription by the PFPC was not assessed. Some studies nevertheless suggest that rapid assessment in triage or the waiting room can lead to over-prescription of radiological examinations [13, 19].

## Conclusion

In conclusion, introducing a PFPC enabled waiting times until first medical assessment to be reduced, as well as SETS-fixed waiting time objectives to be achieved more often. This intervention did neither require additional resources nor impact the ED length of stays or the number of patients leaving without being seen.
